# C-Kit Expression, Angiogenesis, and Grading in Canine Mast Cell Tumour: A Unique Model to Study C-Kit Driven Human Malignancies

**DOI:** 10.1155/2014/730246

**Published:** 2014-05-12

**Authors:** Rosa Patruno, Ilaria Marech, Nicola Zizzo, Michele Ammendola, Patrizia Nardulli, Claudia Gadaleta, Marcello Introna, Gennaro Capriuolo, Rosa Angela Rubini, Domenico Ribatti, Cosmo Damiano Gadaleta, Girolamo Ranieri

**Affiliations:** ^1^Animal Health Unit, Department of Prevention, ASL BAT, Via Andria 176, 70051 Barletta, Italy; ^2^Interventional Radiology Unit with Integrated Section of Translational Medical Oncology, National Cancer Research Centre, “Giovanni Paolo II”, Via Orazio Flacco 65, 70124 Bari, Italy; ^3^Chair of Pathology, University of Bari, Via Casamassima, 70010 Valenzano, Italy; ^4^Chair of Clinical Surgery, University of Catanzaro, Via Europa, 88100 Germaneto, Italy; ^5^Pharmacy Unit, National Cancer Research Centre, “Giovanni Paolo II”, Via Orazio Flacco 65, 70100 Bari, Italy; ^6^Department of Basic Medical Sciences, Neurosciences and Sensory Organs, University of Bari, Piazzale Giulio Cesare 11, 70124 Bari, Italy

## Abstract

Canine cutaneous mast cell tumour (CMCT) is a c-Kit driven tumour sharing similar c-Kit aberrations found in human gastrointestinal stromal tumour. CMCT is classified into three forms: well- (G1), intermediately (G2) (more benign diseases), and poorly (G3) differentiated (malignant) forms. We assess a correlation between c-Kit status, grading, and angiogenesis in CMCTs to explore their potential significance in humans. C-Kit receptor (c-KitR) expression, microvascular density (MVD), and mast cell granulated and degranulated status density (MCGD and MCDD, resp.) were analyzed in 97 CMCTs, by means of histochemistry, immunohistochemistry double staining, and image analysis system. Data showed that predominantly diffuse cytoplasmic- and predominantly focal paranuclear- (Golgi-like) c-Kit protein (PDC-c-Kit and PFP-c-Kit, resp.) expression correlate with high MVD, G3 histopathological grade, and MCDD. Moreover, predominant cell membrane-c-KitR (PCM-c-KitR) expression status correlates with low MVD, G1-G2 histopathological grade, and MCGD. These findings underline the key role of c-Kit in the biopathology of canine MCTs, indicating a link between aberrant c-Kit expression, increased angiogenesis, and higher histopathological grade. CMCT seems to be a model to study contributions of c-Kit activated MCs in tumour angiogenesis and to evaluate the inhibition of MCs activation by means of c-Kit tyrosine kinase inhibitors, currently translated in humans.

## 1. Introduction 

The c-Kit is a protooncogene that encodes for c-Kit receptor (c-KitR), a type III tyrosine kinase protein that is the receptor for stem cell factor (SCF), a cytokine regulating important mast cell (MC) functions, such as growth, differentiation, proliferation, and degranulation [1, 2]. The c-KitR consists of an extracellular domain of 5 immunoglobulin-like folds and an intracellular kinase domain separated by transmembrane and juxtamembrane domains [3]. It is expressed by MCs and their progenitors, by germ cells, and by Cajal interstitial cells [4]. Aberrations of c-Kit, including mutations, deletions, and duplications, have been characterized in human malignancies, such as gastrointestinal stromal tumours (GISTs), mastocytosis, and mast cell leukemia, and in cutaneous canine mast cell tumours (CMCTs) [5–7]. The main effect of these c-Kit aberrations results in a constitutive activation of c-KitR. Thus, they seem to be implicated in both the development and the progression of CMCT that is a very common cutaneous tumour in dog [8]. CMCT is classified in three subgroups: well- and intermediately differentiated (G1 and G2) ones, corresponding to a more benign disease, and poorly differentiated (G3) one, corresponding to a malignant disease which metastasizes to lymph nodes, liver, spleen, and bone marrow; therefore, it is characterized by short overall survival [4]. Preliminary data suggest that G3 CMCT is associated with a higher angiogenic activity as compared to G1 and G2 CMCT [9]. It has been also demonstrated that human and canine MCs play an important role in tumour angiogenesis by means of angiogenic cytokines such as vascular endothelial growth factor (VEGF), platelet derived growth factor (PDGF), fibroblast growth factor-2 (FGF), and tryptase stored in their cytoplasmic secretory granules [10–12]. MCs c-Kit activation leads to several important biological effects, including degranulation, proliferation, survival, decreased apoptosis, and cell adhesion [1, 3]. Recently, a novel tyrosine kinase inhibitor, named masitinib, that targets c-KitR has been developed to treat CMCT, with the aim of translating this approach in human clinical trials [13–16].

According to these lines of evidence, CMCT is an interesting spontaneous tumour model to evaluate the biopathology significance of c-Kit protein expression status and the correlation with angiogenic activities and grading [4, 9]. In this study, we have evaluated c-KitR expression status, microvascular density (MVD), MC granulated and degranulated status density (MCGD and MCDD), and, finally, tumour grading in a series of 97 CMCTs. Interestingly, we have correlated these parameters to each other, by means of histochemistry, immunohistochemistry, double staining, and image analysis methods.

## 2. Material and Methods 

### 2.1. Histochemistry

A series of formalin-fixed and paraffin-embedded tissue samples obtained from 97 cases of CMCTs were utilized. Histological diagnosis was performed on serial slides for each tumour sample stained with haematoxylin-eosin and the Undritz method (Merck, Darmstadt, Germany), specific for red-blue metachromatic MCs identification and granulated/degranulated status [17]. According to Patnaik et al. [18], the cases were classified as follows: 36 were G1, corresponding to well-differentiated CMTC, 29 were G2, corresponding to intermediately differentiated CMTC, and 32 were G3, corresponding to poorly differentiated CMTC.

For the evaluation of c-KitR expression and MVD, three-layer biotin-avidin-peroxidase system, as previously described, was adopted [19]. Briefly, 6 serial sections, for each tissue sample, were cut. After heating, slides were incubated with the rabbit polyclonal antibodies anti-CD117-c-KitR (Dako, Glostrup, Denmark) and with anti-factor VIII-related antigen (FVIII-RA) (Dako, Glostrup, Denmark), used as an endothelial marker [17, 20]. The bound antibodies were visualized by using biotinylated secondary antibody, avidin-biotin peroxidase complex, and 3-amino-9-ethylcarbazole (Dako, Glostrup, Denmark) [20]. Nuclear counterstaining was performed, for each tissue sample, with Gill's haematoxylin (Polysciences, Warrington, PA, USA) [20].

### 2.2. Double Staining

A double stain was also performed by using anti-FVIII-RA antibody and the Undritz method to mark on the same slide both endothelial cells and MCs. As a negative immunohistochemical control, no primary antibody was added.

### 2.3. Image Analysis

The slides were morphometrically evaluated by using an image analysis system (Quantimet 500 Leica). Ten most vascularized areas (hot spot) were selected at low magnification and single red-brown stained endothelial cells, endothelial cell clusters, and microvessels, clearly separated from adjacent microvessels, tumour cells, and other connective tissue elements and MCG/D were counted at ×400 fields in a 0.19 mm^2^ area. In the serial sections c-KitR immunostained cells, MCGD, and MCDD were counted. Hot spots were also observed at ×1000 fields in oil and details were recorded [9, 21].

### 2.4. Statistical Analysis

Mean value ± standard deviations (s.d.) were evaluated for MVD, MCGD, and MCDD in G1, G2, and G3 CMCTs subgroups. The significance of differences in MVD, MCGD, and MCDD between G1 versus G2, G2 versus G3, and G3 versus G1 tumour groups was performed by Student's *t*-test. Correlations between each other among MVD, MCGD, MCDD, predominantly diffuse cytoplasmic-c-KitR (PDC-c-KitR) expression, focal paranuclear- Golgi-like (PFP-c-KitR) expression, and predominant cell membrane-c-KitR (PCM-c-KitR) expression were calculated using Pearson's (r) analysis. All statistical analyses were performed with the SPSS statistical software package (SPSS, Inc., Chicago, Illinois).

## 3. Results

No significant difference was found between G1 and G2 CMCTs subgroups as concerns MVD, MCGD, and MCDD (Table 1). Otherwise, MCDD was significantly higher in G3 (107 ± 42 s.d.) compared to G1 (21 ± 10 s.d., *P* = 0.000) or G2 (24 ± 11 s.d., *P* = 0.000) (Table 1).

**Figure 1:**
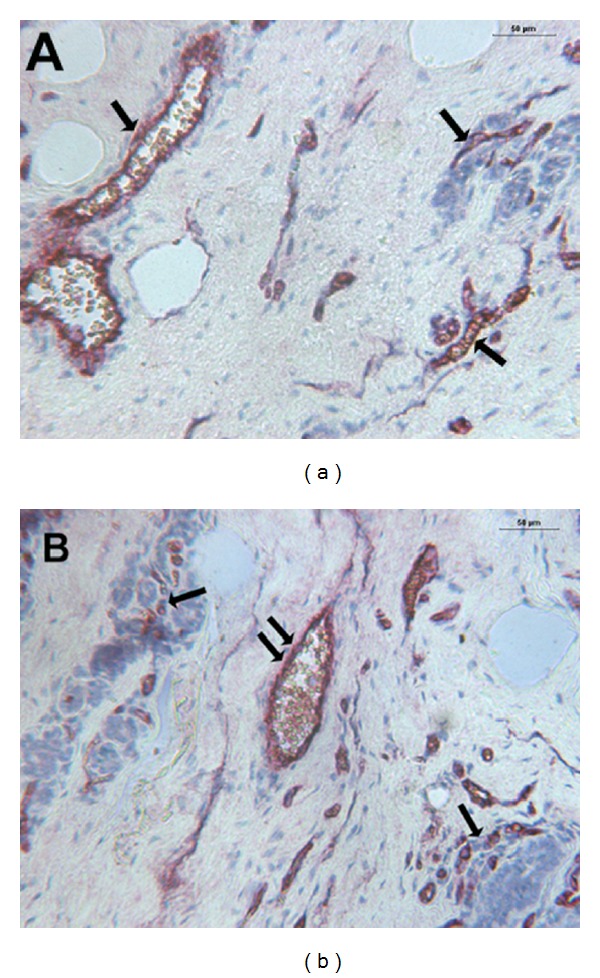
(a) CMCT with a low MVD. Double staining is performed combining immunohistochemistry with toluidine blue histochemistry. Single arrows indicate blood vessels red-brown immunostained with primary anti-factor VIII-related antigen (FVIII-RA) at ×200 magnification. (b) CMCT with a high MVD. Double staining is performed combining immunohistochemistry toluidine blue histochemistry. Single arrows indicate clusters of blue stained neoplastic mast cells, while double arrows indicate blood vessels red-brown immunostained with a primary anti-FVIII-RA at ×200 magnification.

**Figure 2:**
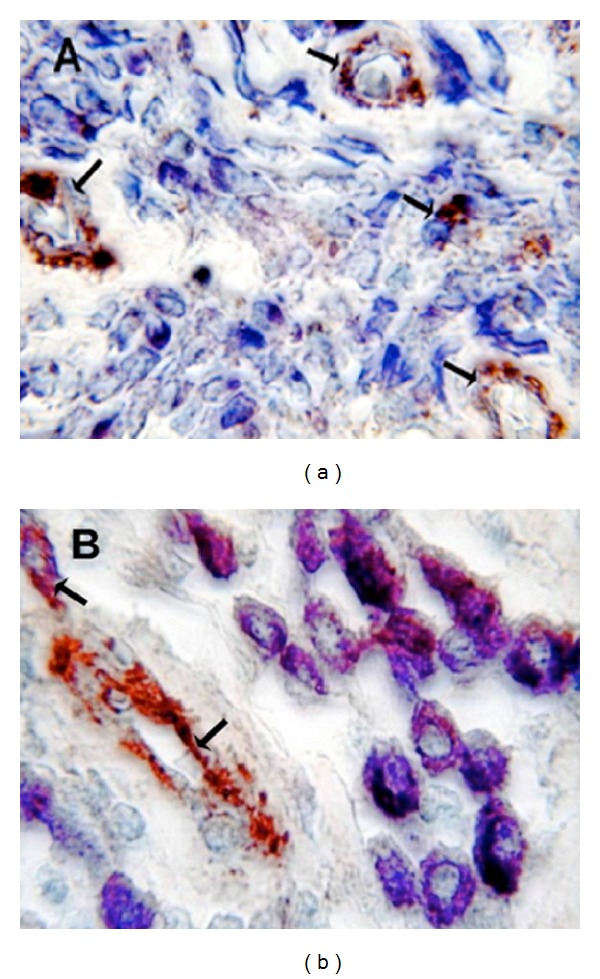
(a) Poorly differentiated G3 CMCT with high MVD. Double staining is performed combining immunohistochemistry with toluidine blue histochemistry. Many scattered degranulated blue stained mast cells. Single arrows indicate red-brown immunostained microvessels with primary anti-FVIII-RA. Note as an internal positive control the red blood cell in the lumen of microvessel. ×1000 in oil magnification. (b) Well-differentiated G1 CMCT with low MVD. Double staining is performed combining immunohistochemistry with toluidine blue histochemistry. Many scattered granulated red-blue stained mast cells. Single arrows indicate red immunostained microvessels with primary anti-FVIII-RA. Note as an internal positive control the red blood cell in the lumen of microvessel. ×1000 in oil magnification.

As concerns MVD, it was significantly higher in G3 compared to G1 or G2 CMCTs subgroups (Figures 1(a) and 1(b) and Table 1). As concerns MCs morphological characteristics, they were often degranulated with less or nonmetachromatic cytoplasmic granules in G3 compared to G1 or G2 CMCTs subgroups in slides stained with both immunohistochemistry and the Undritz method (Figures 2(a) and 2(b)). Furthermore, MCs were often clustered near to or around microvessels in G3 compared to G1 or G2 CMCTs subgroups.

**Figure 3:**
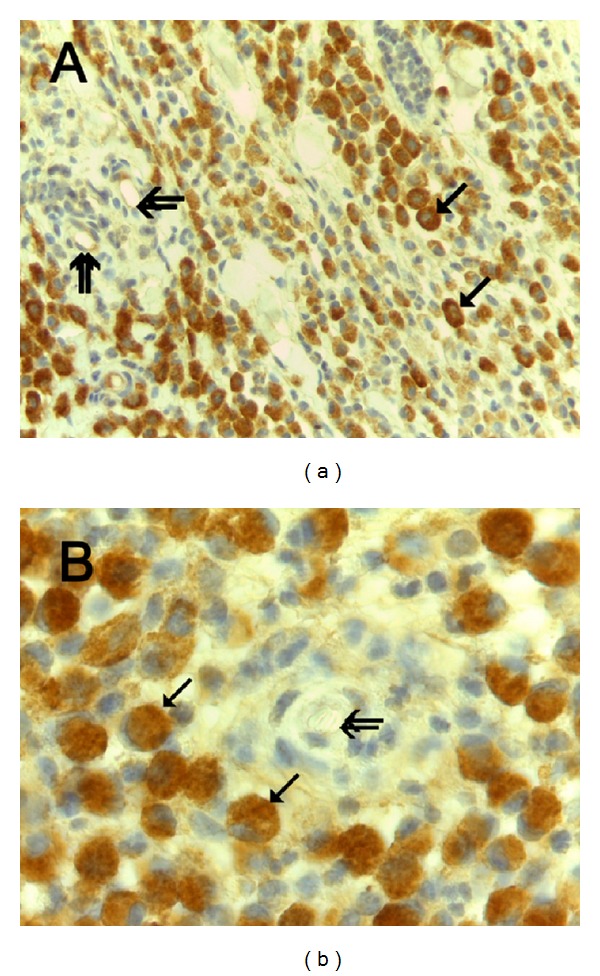
(a) Predominantly diffuse cytoplasmic-c-Kit protein (PDC-c-Kit) expression. Immunohistochemistry is performed with primary anti-c-KitR antibody. Single arrows indicate full brown diffuse cytoplasmic immunostained c-KitR. Double arrows indicate microvessels. ×400 magnification. (b) Particular of (a) at even more magnification. Single arrows indicate full brown diffuse cytoplasmic immunostained c-KitR. Double arrows indicate a microvessel. Note as an internal positive control the red blood cells in the lumen of the microvessel. ×1000 in oil magnification.

**Figure 4:**
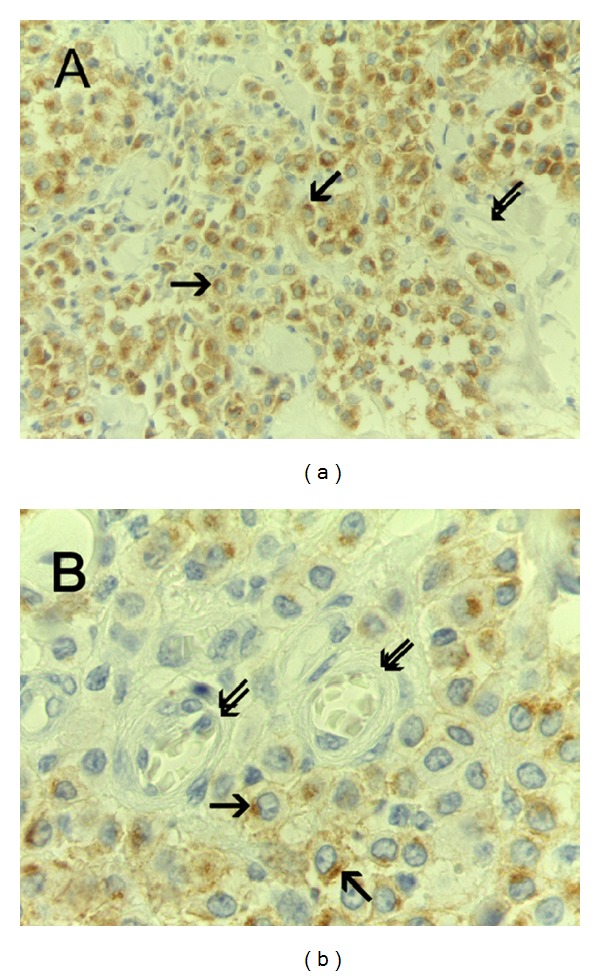
(a) Predominantly focal paranuclear- (Golgi-like) c-Kit protein (PFP-c-Kit) expression. Immunohistochemistry is performed with primary anti-c-KitR antibody. Single arrows indicate focal brown paranuclear cytoplasmic immunostained c-KitR. Double arrows indicate a vessel. Note as an internal positive control the red blood cells in the lumen of the vessel. ×400 magnification. (b) Particular of (a) at even more magnification. Immunohistochemistry is performed with primary anti-c-KitR antibody. Single arrows indicate brown paranuclear cytoplasmic (Golgi-like) immunostained c-KitR. Double arrows indicate microvessels. Note as an internal positive control the red blood cells in the lumen of microvessels. ×1000 in oil magnification.

**Figure 5:**
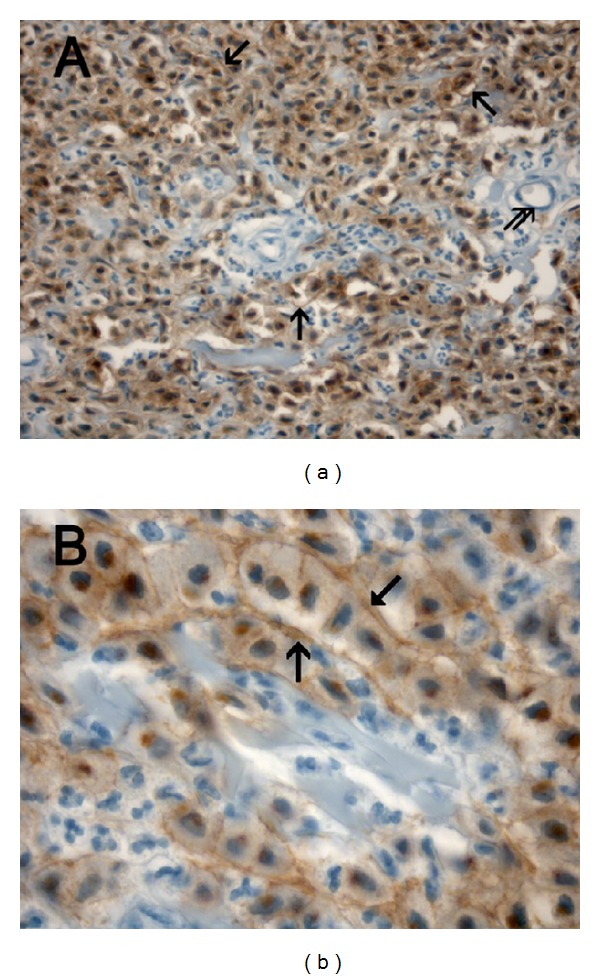
(a) Predominant cell membrane-c-KitR (PCM-c-KitR) expression. Immunohistochemistry is performed with primary anti-c-KitR antibody. Single arrows indicate threadlike cell membrane brown immunostained c-KitR. Double arrows indicate a microvessel. Note as an internal positive control a red blood cell in the lumen of the microvessel. ×400 magnification. (b) Particular of (a) at even more magnification. Immunohistochemistry is performed with primary anti-c-KitR antibody. Single arrows indicate cell membrane brown immunostained c-KitR. ×1000 in oil magnification.

With special references to c-KitR expression status, three patterns of immunostaining were observed: PDC-c-KitR (Figures 3(a) and 3(b)), PFP-c-KitR (Figures 4(a) and 4(b)), and PCM-c-KitR (Figures 5(a) and 5(b)).

**Figure 6:**
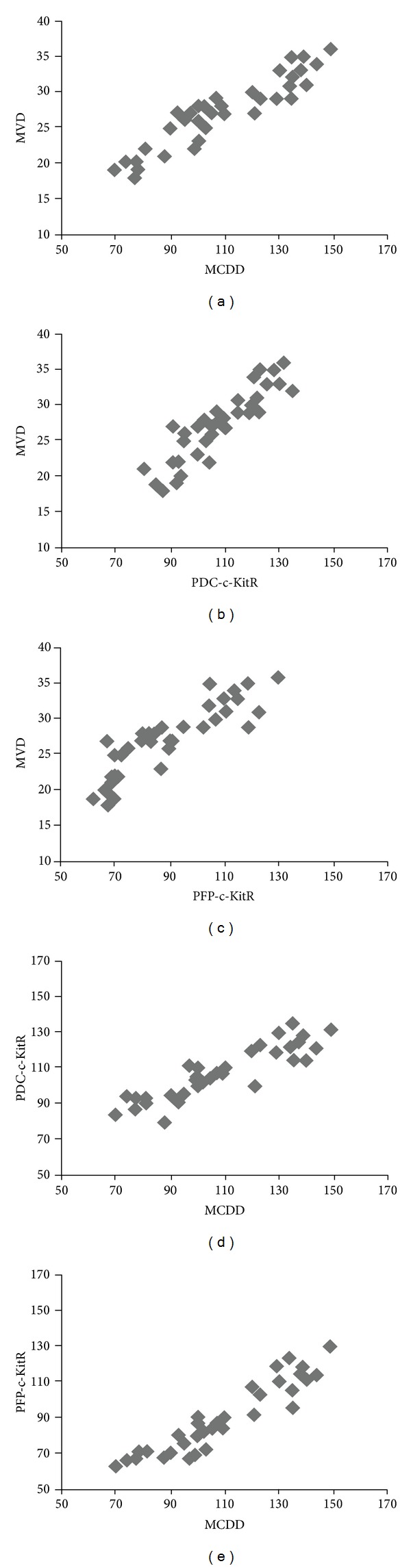
Panel shows the correlations between MVD, MCDD, PFP-c-KitR, and PDC-c-KitR by Pearson's (r) analysis in G3 CMCTs subgroup.

**Figure 7:**
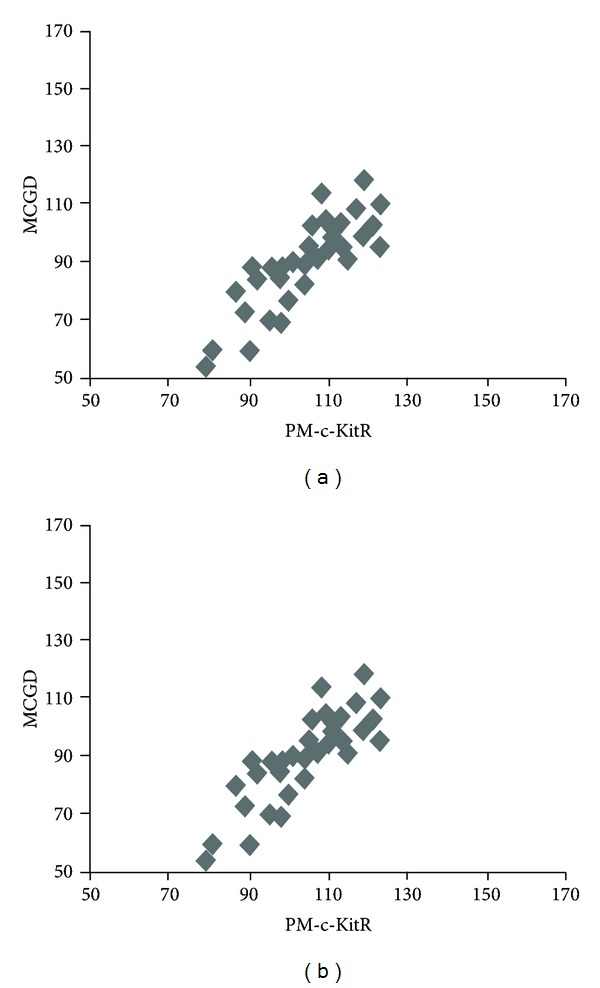
Panel shows the correlations between MCGD and PM-c-KitR by Pearson's (r) analysis in G1 and G2 CMCTs subgroups.

A significant correlation has been established between these parameters: MVD and MCDD (*r* = 0.91, *P* = 0.001), MVD and PDC-c-KitR (*r* = 0.86, *P* = 0.001), MVD and PFP-c-KitR (*r* = 0.83, *P* = 0.001), MCDD and PDC-c-KitR (*r* = 0.90, *P* = 0.001), and MCDD and PFP-c-KitR (*r* = 0.92, *P* = 0.001) in G3 CMCT subgroup (Figure 6) and PM-c-KitR and MCGD in G1 and G2 CMCT subgroups (*r* = 0.78, *P* = 0.002; *r* = 0.75, *P* = 0.002, resp.) (Figure 7).

## 4. Discussion

This is the first report that demonstrates the relationship between c-KitR expression status, MVD, MCGD, and MCDD in regulating tumour angiogenesis and progression of CMCT spontaneous model. MCs involvement in tumour angiogenesis has been demonstrated in several human solid and haematological malignancies [22–29]. MCs can secrete many proangiogenic factors, including FGF-2, tumour necrosis factor alpha (TNF-*α*), interleukin-8 (IL-8), transforming growth factor beta (TGF-*β*), VEGF, and tryptase [30–32]. C-KitR activation leading to MCs degranulation strongly links MCs to angiogenesis [33, 34]. On the other hand, activated c-KitR has been implicated in the pathogenesis of multiple human malignancies; moreover, c-kit aberrations, leading to a constitutively activated form of c-KitR in the absence of its ligand, have been identified in several angiogenesis-dependent diseases, such as GISTs [35, 36], mastocytosis [37], acute myeloid leukemia [38], small cell lung cancer [39], and prostate cancer [40]. Interestingly, similar c-Kit aberrations have been found in CMCTs, also [41–43].

In this study, we have demonstrated that PDC-c-KitR and PFP-c-KitR expression are associated with MCDD and higher MVD in G3 CMCTs subgroup. On the contrary, we have, also, observed that PCM-c-KitR membrane expression is correlated with MCGD and lower MVD in G1 and G2 CMCTs subgroups. We suggest that a possible explanation of the predominant c-KitR cytoplasmic expression is due to c-Kit gene aberrations already demonstrated for G3 CMCT that in turn lead to an abnormal maturation, trafficking, and, finally, cytoplasmic c-Kit protein accumulation [36, 44]. Concerning similar c-Kit oncogene aberrations, which also characterize human GIST, an altered c-KitR protein is transcribed and, consequently, its accumulation occurs in the endoplasmic reticulum, Golgi apparatus, and cytoplasm generating different well-known patterns of c-KitR immunostaining [45, 46]. With particular references to G3 CMCTs, after the c-KitR activation, neoplastic MCs may acquire a survival advantage in terms of proliferation and inhibition of apoptosis, due to the release of angiogenic factors (VEGF, PDGF, FGF-2, and tryptase) contained in their secretory granules that, in turn, stimulate angiogenesis [47–51].

Overall, these data suggest that c-KitR cytoplasmic immunostaining may be a surrogate marker of aggressive behaviour of CMCTs and that it may be an interspecies suitable angiogenic marker of c-KitR activation [11, 45, 49, 52].

Therefore, CMCT seems to be a useful model to study the role of c-Kit activated MCs in tumour angiogenesis.

In conclusion, comparative studies evaluating c-Kit driven human malignancies may be useful to study the inhibition of MCs degranulation or activation by means of novel c-Kit tyrosine kinase inhibitors, such as masitinib (specifically approved for the therapy of CMCTs [7, 15, 16]) that is currently under investigation in human clinical trials ([53, 54], ClinicalTrials.gov identifier: NCT00812240).

## Figures and Tables

**Table 1:** All tissue indexes analysed means ± standard deviations as a function of tumour malignancy grade and statistical significance of their changes between G1 versus G2, G1 versus G3, and G2 versus G3 CMCT groups by Student's *t*-test.

CMCTs (number of cases)	MVD 400x (0.19 mm^2^)	MCGD 400x (0.19 mm^2^)	MCDD 400x (0.19 mm^2^)	PM-c-KitR expression (0.19 mm^2^)	PDC-c-KitR expression (0.19 mm^2^)	PFP-c-KitR expression (0.19 mm^2^)
G1 (36)	7 ± 4	91 ± 29	21 ± 10	109 ± 35	9 ± 4	8 ± 5
G2 (29)	9 ± 5	84 ± 33	24 ± 11	99 ± 24	11 ± 4	10 ± 6
G3 (32)	27 ± 9	39 ± 17	107 ± 42	7 ± 3	115 ± 31	96 ± 34
*t*-test	G1 versus G2	G1 versus G2	G1 versus G2	G1 versus G2	G1 versus G2	G1 versus G2
*P* value	*n.s. *	*n.s*.	*n.s. *	*n.s. *	*n.s. *	*n.s. *
*t*-test	G1 versus G3	G1 versus G3	G1 versus G3	G1 versus G3	G1 versus G3	G1 versus G3
*P* value	*0.001 *	*0.004 *	*0.001 *	*0.000 *	*0.001 *	*0.000 *
*t*-test	G2 versus G3	G2 versus G3	G2 versus G3	G2 versus G3	G2 versus G3	G2 versus G3
*P* value	*0.002 *	*0.004 *	*0.002 *	*0.000 *	*0.001 *	*0.001 *
